# Age-stratified Association Between Plasma Adiponectin Levels and Mortality in Septic Patients

**DOI:** 10.5811/westjem.35607

**Published:** 2025-05-19

**Authors:** Hui Wang, Ming Ma, Jingfeng Dong, Jun Duan

**Affiliations:** * China-Japan Friendship Hospital, Department of Intensive Care Unit, Beijing, People’s Republic of China; † Beijing Haidian Hospital, Department of Orthopedics (Minimally Invasive Spine Surgery Branch), Beijing, People’s Republic of China

## Abstract

**Background:**

Plasma adiponectin (APN) levels might be affected by age. In this study we aimed to study the association between plasma APN levels and age and the effects of APN levels on mortality in age-stratified septic patients.

**Methods:**

We conducted this single-center, retrospective study with 173 patients with sepsis and 57 controls. Physical and demographic characteristics were recorded, and blood samples were collected to measure plasma APN levels. Using this data, we determined the association between plasma APN levels and age, and the effect of plasma APN levels on mortality in age-stratified septic patients.

**Results:**

We stratified patients into three age groups: < 60 years (middle age); 60–80 years (advanced age); and elderly (≥ 80 years). Plasma APN levels increased with increasing age in both the control group and the sepsis group. Mortality also increased with age: 12.3% in the < 60 group; 24.6% in those 60–80 years of age; and 36.2% in elderly patients >80 years (P<0.001). In middle-aged and advanced-age patients, APN levels were found to be associated with 28-day mortality based on the receiver operating characteristic curve analysis. Furthermore, APN levels remained independently associated with 28-day mortality in patients < 80 years. However, in elderly patients the APN levels showed no significant association with 28-day mortality.

**Conclusion:**

We found a positive association between plasma adiponectin levels and age in septic patients. Low circulating levels of APN were associated with 28-day mortality in septic patients < 80 years of age. We found no significant association between APN and mortality in sepsis patients who were > 80 years of age.

## INTRODUCTION

In recent years, the growing elderly population has contributed to a rise in intensive care unit (ICU) admissions and increased mortality among critically ill older patients.^1^–^3^ Demographic changes have made older adults a significant percentage of ICU patients, with multiple comorbidities complicating their clinical status and making them particularly vulnerable to infection-related complications. Consequently, many critical care studies have increasingly focused on this demographic.

Adiponectin (APN), a peptide secreted by adipose tissue, enhances insulin sensitivity and regulates lipid metabolism.^4^,^5^ Reduced APN activity is linked to metabolic syndrome, including obesity, type 2 diabetes, and cardiovascular diseases.^6^,^7^ Beyond its metabolic roles, APN also has anti-inflammatory effects.^8^,^9^ Studies have shown a negative correlation between APN, pro-inflammatory cytokines, illness severity, and mortality in septic patients.^10^,^11^ Our previous research similarly found that low APN levels were associated with 28-day mortality in sepsis patients.^12^

Reports suggest age is associated with elevated plasma APN levels in healthy adults, despite increased cardiovascular risk in the elderly.^13^–^16^ This age-related increase may be linked to visceral fat^15^ and renal function,^14^,^16^ although it is less clear in pathological conditions such as type 2 diabetes.^17^ Given the uncertainty with regard to sepsis, we aimed to explore the relationship between plasma APN levels, age, and sepsis-related mortality across different age groups.

## METHODS

### Population and Protocol

We conducted a retrospective chart review of ICU patients from January 2019–January 2022, following recommended methodological standards for retrospective studies.^18^ The study was approved by the ethics review board, and informed consent was obtained from patients or their families if the patient was mechanically ventilated or had altered mental status. We included a total of 173 sepsis patients (>18 years of age) admitted to the ICU for more than 48 hours. Sepsis was defined according to consensus international guidelines as life-threatening organ dysfunction that is caused by dysregulation of the host response to infection.^19^ The exclusion criteria included pregnancy, malignancy, and non-sepsis-related immunosuppression. Fifty-seven postoperative patients without sepsis served as the control group.

This study adheres to the STROBE (Strengthening the Reporting of Observational Studies in Epidemiology) guidelines for observational studies.^20^

### Data Collection

Baseline clinical and laboratory characteristics included age, sex, past illness, and blood chemistry (eg, brain natriuretic peptide [BNP], C-reactive protein [CRP], procalcitonin, serum creatinine, alanine transaminase, high-sensitivity troponin T, and arterial blood gas) were measured using standard clinical methods at the China-Japan Friendship Hospital. Data was collected at the time of a diagnosis of sepsis or upon admission. Disease severity was assessed by Sequential Organ Failure Assessment (SOFA) scores and Acute Physiology and Chronic Health Evaluation II (APACHE-II) scores on the day of diagnosis.

### Plasma Adiponectin Levels and Biochemical Assays

Blood samples were obtained on the day of admission or sepsis diagnosis and centrifuged at 1000×g for 15 minutes at 4°C within 30 minutes of collection. Plasma was withdrawn and stored at −80°C until analysis. Plasma APN levels were measured using an enzyme-linked immunosorbent assay (ELISA) for human APNs (human APN ELISA kit; R&D Systems, Emeryville, CA). Optical density was measured at 450 nanometers. The limit of APN detection was 0.891 nanograms per milliliter according to the manufacturer’s instructions.

**Population Health Research Capsule**

What do we already know about this issue?

*Plasma adiponectin (APN) levels may increase with age and influence mortality in septic patients, but age-specific effects remain unclear.*

What was the research question?

*How do plasma APN levels correlate with age and mortality in age-stratified septic patients?*

What was the major finding of the study?

*The APN levels predicted 28-day mortality in patients < 80 years (P < 0.001), but no association was found in patients ≥ 80 years.*

How does this improve population health?

*Identifying APN as a mortality predictor in sepsis patients < 80 might aid in early intervention and tailored treatment, possibly improving outcomes and survival rates.*

### Statistical Analysis

We analyzed data using SPSS 23.0 (SPSS Statistics, IBM Corp, Armonk, NY) and Prism 7.0 (GraphPad, Inc, San Diego, CA). The Kolmogorov-Smirnov test assessed normality. Continuous variables are presented at mean ± SD or median (25th–75th percentile), while categorical variables are shown as numbers and percentages. We performed group comparisons using unpaired *t*-tests (normal distribution) or Mann-Whitney U tests (non-normal distribution). Sepsis patients were divided into three age groups (<60, 60–80, and ≥80 years), and we compared variables using analysis of variance (ANOVA) or Kruskal-Wallis H tests. Pearson or Spearman correlation coefficients assessed associations between APN and various parameters. Kaplan-Meier curves were used to analyze survival across age groups. Logistic regression identified risk factors for sepsis-related mortality, reported as odds ratios (OR) and 95% confidence intervals (CI). Receiver operating characteristic (ROC) curves determined the sensitivity and specificity of predicting 28-day mortality in sepsis. A *P*-value of <0.05 was considered statistically significant.

Sample size calculation was based on previous results showing that the mortality rates for the age groups were estimated as follows: <60 years (11%); 60–80 years (14.2%); and >80 years of age (18%).^21^,^22^ We used G*Power software (Heinrich Heine University, Düsseldorf, Germany) and performed an ANOVA for three independent groups. The total required sample size for a one-way ANOVA was 144 patients with 85% power, an alpha level of .05, and an effect size of .28. Assuming a rate of 20% for missing data or incomplete follow-up, we decided to include 173 patients.

## RESULTS

### Clinical Characteristics of Patients

In the sepsis or septic shock group, we included an initital total of 182 patients. Of them, five were excluded because they were already in a terminal condition upon ICU admission, and four were excluded because of long-term use of steroids. As a result, 173 sepsis patients were included with a mean age of 67.58±14.07 years. According to previous studies, age is closely associated with sepsis mortality.^22^,^23^ Research has shown that patients ≥80 years of age have a higher in-hospital mortality rate compared to those 65–79 years of age.^23^ Therefore, based on previous studies, we stratified the included patients into three age groups: <60 years (middle-aged), 60–80 years (advanced age), and >80 years (elderly). The main sources of sepsis were gastrointestinal (38.7%), pulmonary (31.8%), cholangitis (16.2%), genitourinary (8.1%), and bloodstream infection (5.2%).

Table 1 summarizes patient characteristics. Hypertension, diabetes, coronary artery disease, and chronic kidney disease were more common in the elderly. Elderly patients also had higher APACHE II scores, serum creatinine, BNP, and CRP. No significant differences were noted between age groups for gender, SOFA scores, and other laboratory parameters listed in Table 1.

### Variation in Adiponectin Levels in Sepsis Patients

**Figure 1:**
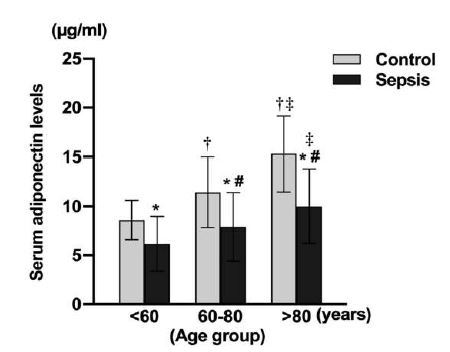
The plasma adiponectin levels in each age group in the control and sepsis groups. *P <0.05 vs the control group in each age group. # P <.05 vs < 60 groups in sepsis patients, ^†^P <.05 vs < 60 groups in controls, ^‡^P <.05 vs 60–80 group both in control and sepsis patients. *μg/mL*, micrograms per mililiter.

**Figure 2:**
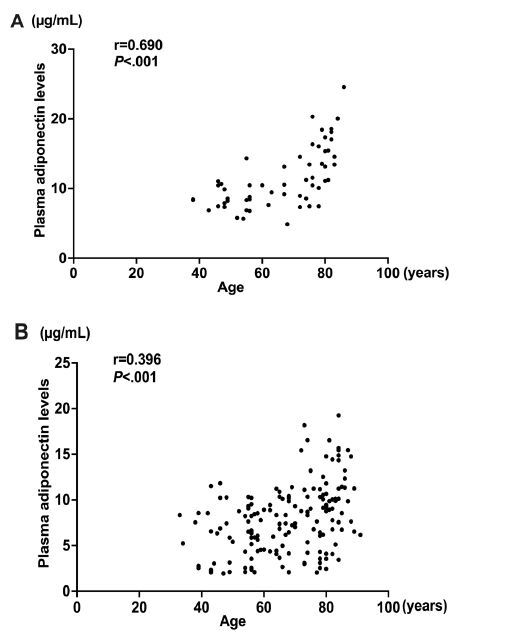
The positive association between age and adiponectin (APN) level in the control (A) and sepsis group (B). Correlation between the plasma APN level and age was confirmed by values of r = 0.690, P <.001 and r = 0.396, P <.001 in the control and sepsis groups, respectively. *μg/mL*, micrograms per mililiter.

Plasma APN levels in each age group are shown in Table 1 and Figure 1. Plasma APN levels were significantly lower in patients with sepsis than those in patients without sepsis in all age groups and increased with age in both groups. The plasma APN level was negatively correlated with age in both the control group and the sepsis group (r = .690, *P*<.001 and r = .412, *P*<.001; Figure 2).

### Comparison of Clinical Outcomes

**Figure 3:**
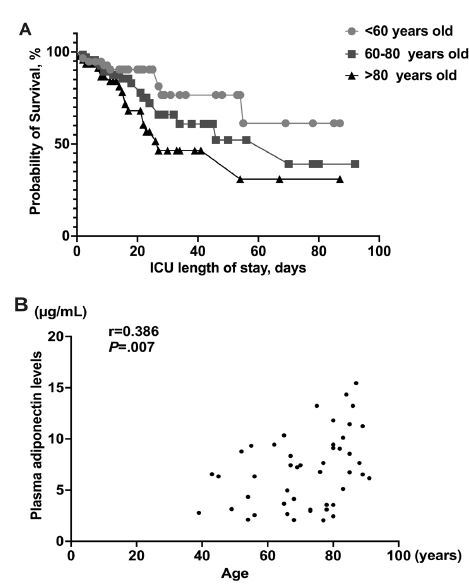
A. Survival curves were generated by the Kaplan-Meier method for middle-aged, advanced, and elderly patients with sepsis. B. The positive association between age and adiponectin (APN) level in deceased patients. Correlation between APN level and age was confirmed by values of r =.386 and P<.001. *ICU*, intensive care unit.

Clinical outcomes across age groups are presented in Table 1. Elderly patients had significantly higher 28-day (36.2% vs 24.6% vs 12.3%, *P*=.01) and in-hospital mortality (38.2% vs 27.5% vs 15.8%, *P*=.03) and required longer mechanical ventilation (15, interquartile range [IQR] 7–27 vs 5 IQR, 2–19 vs 4, IQR 1–9) compared to advanced-age and middle-aged patients. Subgroup analysis of 28-day mortality is detailed in Figure 3 and Table 2. Survival was significantly lower in older age groups (Figure 3A). In deceased patients, elderly patients had higher plasma APN levels, which were negatively correlated with age (r=.386, *P*=<.01; Table 2, Figure 3B).

### Association Between Adiponectin and Mortality in Different Age Stratifications

We found that both in-hospital mortality and APN levels increased with age, unlike previous studies associating low APN levels with sepsis mortality.^12^ To investigate further, we analyzed the link between APN levels and 28-day mortality by age group. Univariate logistic regression (Table 3) showed that low APN levels were associated with higher 28-day mortality in patients < 60 years and 60–80 years. In these groups, APACHE II and SOFA scores were also significant, along with serum creatinine, BNP, and CRP in the 60–80 group. In patients > 80 years, only APACHE II scores and BNP levels were significantly related to 28-day mortality, with no association found for APN.

The multivariate regression analysis of significant parameters revealed that among patients < 60 years of age, only APN and APACHE II scores were independently associated with 28-day mortality. In the 60–80 years group, APN, APACHE II, and BNP were also significant independent predictors of 28-day mortality (Table 4).

**Figure 4:**
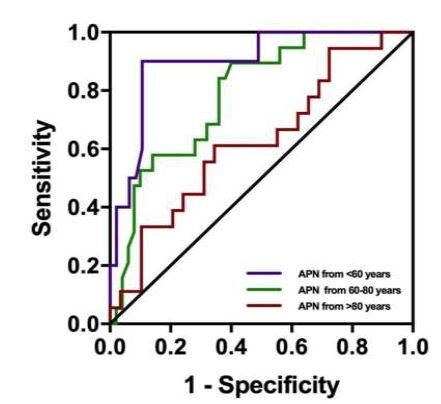
Receiver operating characteristic curve analysis of the association of adiponectin with 28-day mortality in septic patients at the age 80 years. *APN*, adiponectin.

We conducted ROC curve analyses to assess the association between APN levels and 28-day mortality across the three age groups. The AUC values, as well as the sensitivity and specificity of APN in association with sepsis related mortality, decreased with age. In elderly patients, APN was not significantly associated with mortality. However, in middle-aged and advanced age patients, APN showed strong discriminative ability (AUC = 0.872 and AUC = 0.774). APN cutoff values of 3.355 μg/ml and 7.985 μg/ml predicted mortality with sensitivities of 88.89% and 83.33%, and specificities of 87.5% and 62.75%, respectively (Table 5, Figure 4).

## DISCUSSION

In this study we found that plasma APN levels were significantly lower in sepsis patients. Both control and sepsis groups exhibited a positive correlation between plasma APN levels and age. Mortality rates were notably higher in patients ≥ 60 years of age, with those ≥ 80 years experiencing even greater mortality. The APN levels were associated with 28-day mortality in patients < 80 years, as indicated by the AUC, where lower APN levels correlated with higher mortality. However, no significant association with 28-day mortality was observed in patients aged ≥ 80 years.

Adiponectins have anti-inflammatory effects and may play a role in acute inflammatory diseases through direct effects on inflammatory cells and interactions with tumor necrosis factor-alpha.^24^ Early data in septic patients indicated decreased APN levels, but it is unclear whether this results from the disease or whether lower levels predispose patients to critical illness.^24^ Soares et al noted that oxidative stress in adipose cells leads to reduced APN secretion and higher lactate levels in sepsis.^25^ Our studies found lower APN levels linked to greater sepsis severity; however, prior research reported conflicting results, showing either increased or similar APN levels compared to controls.^26^,^27^ These discrepancies could stem from differences in study design, sepsis stages, and patient characteristics, such as age, gender, and exclusion criteria.^28^

Several studies show a correlation between APN levels and survival in sepsis patients. APN levels showed a stronger association with 28-day survival than other factors, including the APACHE II score.^12^,^26^ Hillenbrand et al found significant changes in plasma adipokine levels in severe sepsis, with higher pro-inflammatory and lower anti-inflammatory factors.^28^ In our study, multivariate regression analysis indicated that 28-day mortality was significantly correlated with APN levels in patients <80 years of age. These findings highlight the potential utility of APN as a biomarker for early risk stratification and therapeutic targeting in sepsis.

A large observational cohort reported higher mortality among patients > 85 compared to the general population.^29^ Additionally, age > 60 was linked to mortality in ICU patients with intra-abdominal infections, and patients ≥80 of age faced the worst prognosis.^30^ Our study also found increased mortality with age, particularly in those ≥ 80. Statistical analysis using multivariate regression models demonstrated that age was an independent predictor of 28-day mortality (*P* < .05). When stratified by age groups (< 60 years, 60–79 years, and ≥ 80 years), the mortality rates were observed to increase progressively, with rates of 12.3%, 24.6%, and 36.2%, respectively. Factors contributing to this increase include altered immunity, chronic diseases, malnutrition, and frailty.^30^ The findings underscore the importance of incorporating age as a critical variable in the risk stratification of sepsis patients. Tailored interventions targeting older adults, including early recognition, aggressive supportive care, and personalized management plans, are essential to mitigate the impact of age-related mortality risk.

We further examined the relationship between APN levels and age in sepsis patients. Previous research indicated increased plasma APN levels in older individuals.^13^–^15^,^31^ Obata et al showed that APN levels positively correlated with age, independent of renal function and body fat.^17^ Consistent with these findings, we observed a statistically significant positive correlation between age and APN levels (r=.386, P=.01). When stratified by age groups, the APN levels showed a stepwise increase, with levels of 0.389 (0.181–0.632) μg/ml, 0.687 (0.540–0.840) μg/ml, 0.882 (0.735–1.036) μg/ml, respectively. The mechanisms behind the age-related increase in APN levels remain unclear. Some studies suggest a link to declining renal function,^14^ while others report no association.^16^ Our findings align with hypotheses that aging-related changes in metabolism and inflammation may play a role in regulating APN levels. Given the known anti-inflammatory and insulin-sensitizing properties of APN, these results highlight its potential involvement in age-related metabolic and inflammatory processes in sepsis.

Although APN levels increase in chronic kidney disease,^32^ type 1 diabetes,^33^ and heart failure,^34^ hypoadiponectinemia remains an independent risk factor due to arteriosclerosis progression. The rise in APN levels with age appears paradoxical, as high APN in the elderly may not provide benefits due to advanced atherosclerosis.^35^ Our findings support this paradoxical relationship. Specifically, multivariate regression analysis indicated that APN levels were significantly associated with 28-day mortality in patients <60 years and 60–80 years of age (OR .296, 95% CI .079–0.599, *P*=.01, and OR .651, 95% CI .411-.907, *P*=.03, respectively), but not in patients ≥80 years. These results suggest that while elevated APN levels may have protective effects in younger sepsis patients, their role in the elderly is likely diminished due to the presence of advanced atherosclerosis and other age-related comorbidities. Further research is needed to clarify APN’s role in elderly sepsis patients, particularly to explore potential interventions targeting APN pathways to improve outcomes.

## LIMITATIONS

Limitations of this study include its single-center design and small sample size, which may affect the generalizability of findings. We also lacked comprehensive longitudinal data with multiple plasma measurements, and selection bias may exist due to missing data, particularly among elderly patients. Larger multicenter trials are needed to better understand APN’s role in sepsis.

## CONCLUSION

We found a significant positive association between plasma adiponectin levels and age in septic patients. Low circulating APN levels were associated with a higher risk of death in septic patients < 80 years of age, serving as an independent predictor of mortality in this group. However, the association between APN and mortality was not significantly higher in patients > 80 years of age. Based on these results, clinicians should consider age-related changes in plasma APN levels when interpreting a patient’s levels in clinical practice.

## Figures and Tables

**Table 1:** Clinical characteristics of patients with sepsis at admission.

	Middle-aged patients (< 60 years) (n=57)	Advanced age patients (60–79) (n=69)	Elderly patients (≥ 80 years) (n=47)	P
Age (years)	54 (45–56)	70 (66–75.5)	84 (81–85)	<.001
Male (n [%])	25 (43.9)	39 (56.5)	26 (55.3)	.32
Body mass index (kg/m2)	23.04 (20.96–24.54)	23.43 (21.6–25.18)	22.89 (19.63–25.28)	.59
Past illness				
Hypertension (n [%])	19 (33.3)	37 (53.6)	32 (68.1)	<.01
Diabetes (n [%])	10 (17.5)	41 (59.4)	36 (76.6)	<.001
Coronary artery disease (n [%])	17(29.8)	31(44.9)	26(55.3)	.02
Chronic kidney disease (n [%])	11(19.3)	29(42.0)	24(51.1)	<.01
Severity of illness				
APACHE II score	16 (11–24)	19 (14–23)	23 (19–28)	<.001
SOFA score	11 (7.5–15)	9 (6–13.5)	10 (7–12)	.36
Source of infection N (%)				
Gastrointestinal	14(24.5)	32(46.4)	22(46.8)	.02
Pulmonary	18(34.0)	18(26.1)	17(36.2)	.46
Cholangitis	14(25.5)	12(17.4)	5(10.7)	.15
Genitourinary	8(14.0)	6(8.7)	2(4.4)	.23
Bloodstream infection	3(5.4)	1(1.5)	1(2.1)	.40
Laboratory parameters				
Glucose (mmol/L)	11.3 (9.4–14.3)	11.7 (9.4–15.4)	10.7 (9.5–12.5)	.25
Serum creatinine (μmol/L)	106.2 (75.7–173.3)	132.2 (75.3–239.5)	174.4 (108–263.5)	<.01
ALT (IU/L)	37 (19.5–76.5)	31 (14–71)	21 (12–81)	.49
Brain natriuretic peptide (pg/mL)	840.5 (302–1442)	1,133 (545–2066)	1,328 (661.9–5053)	<.01
hsTnT (ng/mL)	0.122 (0.024–0.482)	0.056 (0.012–0.486)	0.192 (0.04–0.643)	.07
Procalcitonin (ng/mL)	6.86 (3.12–20.75)	11.96 (3.73–24.85)	8.71 (1.08–33.4)	.19
CRP (mg/L)	24.24 (13.51–55.2)	45 (15.8–96.13)	76.18 (45.26–145)	<.001
Lactic acid (mmol/L)	3.2 (1.9–4.75)	2.6 (1.6–3.9)	2.3 (1.9–4.4)	.22
APN (μg/mL)	6.34 (3.35–8.49)	8.32 (4.77–10.34)	9.87 (7.58–12.33)	<.001
Clinical outcomes				
Invasive ventilation duration (d)	4 (1–9)	5 (2–19)	15 (7–27)	<.001
28-day mortality (n [%])	7 (12.3)	17 (24.6)	17 (36.2)	.01
In-hospital mortality (n [%])	9 (15.8)	19 (27.5)	18 (38.2)	.03

The data are expressed as a median with interquartile range or number of patients.

*APACHE II*, Acute Physiology and Chronic Health Evaluation II; *SOFA*, Sequential Organ Failure Assessment; *ALT*, alanine transaminase; *hsTnT*, high-sensitivity troponin T; *CRP*, C-reactive protein; *APN*, adiponectin; *IQR*, interquartile range.

**Table 2:** Comparison of adiponectin levels in sepsis patients of different ages according to the patient outcome.

Patients with sepsis	APN (μg/mL)			

	< 60 years	60–80 years	> 80 years	*P*
Surviving patients	6.53 (4.4–8.54)	8.91 (6.19–10.45)	9.9 (8.28–14.6)	<.001
Deceased patients	4.98 (3.1–7.65)	5.34 (2.73–9.33)	9.08 (6.45–11.53)	<.01

*APN*, adiponectin.

**Table 3:** Univariate logistic regression analysis of factors associated with 28-day mortality in different age stratifications.

	< 60 years			60–80 years			> 80 years		
		
	OR	95% CI	P	OR	95% CI	P	OR	95% CI	P
Sex	.825	.189–3.274	.79	.805	.276–2.356	.69	1.467	.448–5.003	.53
Body mass index	1.032	.791–1.368	.82	1.024	.839–1.254	.82	0.979	.813–1.176	.83
Hypertension	.830	.162–3.442	.29	1.269	.439–3.787	.66	1.368	.388–5.258	.63
Diabetes	.756	.148–3.122	.71	2.800	.918–9.743	.08	0.678	.171–2.763	.58
Coronary artery disease	2.917	.702–12.29	.13	2.063	.712–6.207	.18	1.467	.448–5.003	.53
Chronic kidney disease	2.089	.388–9.389	.37	2.593	0.879–7.843	.08	0.650	.194–2.115	.47
Glucose	0.889	.709–1.068	.22	.984	0.862–1.114	.80	1.030	.842–1.259	.77
APACHE II	1.122	1.109–1.253	.02	1.234	1.115–1.414	<.001	1.121	1.027–1.242	.02
SOFA	1.221	1.040–1.480	.02	1.158	1.023–1.326	.03	1.219	1.047–1.456	.02
Serum creatinine	1.012	1.004–1.022	.01	1.007	1.001–1.013	.01	0.997	0.991–1.001	.18
Alanine transaminase	1.001	.991–1.008	.84	.998	0.991–1.003	.48	1.001	0.999–1.004	.11
BNP	.649	.236–1.364	.28	1.780	1.133–2.950	.01	2.165	1.359–3.858	<.001
hsTnT	.842	.227–1.931	.73	1.305	0.809–2.089	.26	3.785	1.21–18.38	.02
Procalcitonin	.983	.927–1.024	.45	.998	.972–1.020	.85	1.000	0.975–1.024	.99
C-reactive protein	1.008	.992–1.022	.29	1.013	1.004–1.022	.004	1.009	0.999–1.019	.08
Lactic acid	1.014	.841–1.158	.85	.985	.788–1.178	.88	1.113	0.886–1.415	.35
Adiponectin	.389	.181–0.632	<.001	.687	.540–.840	<.001	.882	.735–1.036	.13
Infection source									
Gastrointestinal	.903	.544–10.46	.22	1.541	.881–7.99	.09	.572	.543–5.961	.35
Pulmonary	.452	.356–6.397	.54	1.622	.640–6.523	.22	.347	.405–4.889	.58

Odds ratios, 95% CIs, and P-values were obtained by univariate logistic regression analysis.

*OR*, odds ratio; *CI*, confidence interval; *APACHE II*, Acute Physiology and Chronic Health Evaluation II; *SOFA*, sequential organ failure assessment; *BNP*, brain natriuretic peptide; *hsTnT*, high-sensitivity troponin T.

**Table 4:** Multivariate logistic regression analysis of factors associated with 28-day mortality in different age stratification.

	< 60 years			60–80 years			> 80 years		
		
	OR	95% CI	P	OR	95% CI	P	OR	95% CI	P
APACHE II	1.24	1.072–1.508	.01	1.282	1.101–1.585	.006	1.186	1.048–1.383	.01
SOFA	1.037	.817–1.316	.76	1.200	.993–1.503	.076			
Serum creatinine				1.009	1.000–1.021	.069			
BNP				3.690	1.656–11.17	.005	1.957	1.167–3.646	.02
C-reactive protein				1.002	.988–1.016	.748			
Adiponectin	.296	.079–.599	.01	0.651	.411–0.907	.027			

Odds ratios, 95% CIs, and P-values were obtained by multivariate logistic regression analysis.

*OR*, odds ratio; *CI*, confidence interval; *APACHE II*, Acute Physiology and Chronic Health Evaluation II; *SOFA*, sequential organ failure assessment; *BNP*, brain natriuretic peptide.

**Table 5:** Receiver operating characteristic analysis of adiponectin levels for predicting 28-day mortality in septic patients across different age groups.

Patients with sepsis	Cutoff value	AUC (95% CI)	Sensitivity	Specificity	P
<60 years	3.355	0.872 (.759–.983)	88.89	87.50	<.001
60–80 years	7.985	0.774 (.658–.890)	83.33	62.75	<.001

The cutoff value was determined using receiver operating characteristic curve analysis.

*AUC*, the area under the curve; *CI*, confidence interval.
